# Neurotrophic factor small-molecule mimetics mediated neuroregeneration and synaptic repair: emerging therapeutic modality for Alzheimer’s disease

**DOI:** 10.1186/s13024-016-0119-y

**Published:** 2016-07-11

**Authors:** Syed Faraz Kazim, Khalid Iqbal

**Affiliations:** Department of Neurochemistry, and SUNY Downstate/NYSIBR Program in Developmental Neuroscience, New York State Institute for Basic Research (NYSIBR), 1050 Forest Hill Road, Staten Island, NY 10314 USA; Graduate Program in Neural and Behavioral Science, and Department of Physiology and Pharmacology, State University of New York (SUNY) Downstate Medical Center, 450 Clarkson Avenue, Brooklyn, NY 11203 USA

**Keywords:** Alzheimer’s disease, Cognition, Neurotrophic factor small-molecule mimetics, Brain-derived neurotrophic factor (BDNF), Ciliary neurotrophic factor (CNTF), Amyloid beta, Tau, Neurogenesis, Synaptic loss, Neurodegeneration

## Abstract

Alzheimer’s disease (AD) is an incurable and debilitating chronic progressive neurodegenerative disorder which is the leading cause of dementia worldwide. AD is a heterogeneous and multifactorial disorder, histopathologically characterized by the presence of amyloid β (Aβ) plaques and neurofibrillary tangles composed of Aβ peptides and abnormally hyperphosphorylated tau protein, respectively. Independent of the various etiopathogenic mechanisms, neurodegeneration is a final common outcome of AD neuropathology. Synaptic loss is a better correlate of cognitive impairment in AD than Aβ or tau pathologies. Thus a highly promising therapeutic strategy for AD is to shift the balance from neurodegeneration to neuroregeneration and synaptic repair. Neurotrophic factors, by virtue of their neurogenic and neurotrophic activities, have potential for the treatment of AD. However, the clinical therapeutic usage of recombinant neurotrophic factors is limited because of the insurmountable hurdles of unfavorable pharmacokinetic properties, poor blood–brain barrier (BBB) permeability, and severe adverse effects. Neurotrophic factor small-molecule mimetics, in this context, represent a potential strategy to overcome these short comings, and have shown promise in preclinical studies. Neurotrophic factor small-molecule mimetics have been the focus of intense research in recent years for AD drug development. Here, we review the relevant literature regarding the therapeutic beneficial effect of neurotrophic factors in AD, and then discuss the recent status of research regarding the neurotrophic factor small-molecule mimetics as therapeutic candidates for AD. Lastly, we summarize the preclinical studies with a ciliary neurotrophic factor (CNTF) small-molecule peptide mimetic, Peptide 021 (P021). P021 is a neurogenic and neurotrophic compound which enhances dentate gyrus neurogenesis and memory processes via inhibiting leukemia inhibitory factor (LIF) signaling pathway and increasing brain-derived neurotrophic factor (BDNF) expression. It robustly inhibits tau abnormal hyperphosphorylation via increased BDNF mediated decrease in glycogen synthase kinase-3β (GSK-3β, major tau kinase) activity. P021 is a small molecular weight, BBB permeable compound with suitable pharmacokinetics for oral administration, and without adverse effects associated with native CNTF or BDNF molecule. P021 has shown beneficial therapeutic effect in several preclinical studies and has emerged as a highly promising compound for AD drug development.

## Background

Alzheimer’s disease (AD) is a chronic, debilitating, neurodegenerative disorder and is the most common form of dementia in the elderly [1, 2]. AD is the sixth leading cause of mortality in the United States, and affects nearly 5.4 million Americans [3, 4]. Approximately 476,000 people age 65 or older are expected to develop AD in the United States in the year 2016, and the total healthcare expenditure for AD and related dementias will be nearly 236 billion dollars, making it one of the costliest chronic diseases in the United States [4]. Worldwide, AD and related dementias affect approximately 47 million people [5]. As yet there is no effective cure for AD [6]. By the year 2050, the prevalence of AD is projected to be 13.8 million in United States [3, 7], and 135 million worldwide [5, 8], lest an effective AD therapy is developed.

AD is clinically characterized by a heterogeneous set of symptoms including progressive memory impairment, visuospatial decline, aphasia, and loss of executive function [1, 9–11]. Short-term memory loss is the most common early symptom of AD [1, 9–11]. Histopathologically, there are two major lesions in AD: senile plaques (diffuse and neuritic) and neurofibrillary tangles (NFTs) [12, 13]. Amyloid β (Aβ) is the main component of the senile plaques whereas abnormally hyperphosphorylated tau protein gives rise to NFTs [12, 13]. Alongside Aβ plaques and NFTs, impairments in adult hippocampal neurogenesis and synaptic plasticity, synaptic deficit, and profound neurodegeneration are also major features of AD [14–25]. Adult hippocampal neurogenesis and synaptic plasticity have been proposed to be essential for learning and memory [26–30]. Alterations in adult hippocampal neurogenesis and synaptic plasticity could thus be the major mechanisms underlying cognitive dysfunction in AD [18, 31]. AD has also been characterized as a consequence of synaptic failure [22, 32]. Several studies have shown that synaptic loss correlates better with cognitive decline than either Aβ plaque load or NFTs [25, 33–35]. Targeting the impairment in adult hippocampal neurogenesis and neuronal and synaptic loss has been proposed as a potential therapeutic approach to rescue cognitive dysfunction in AD [6, 36].

## Main text

### Etiopathogenesis of Alzheimer’s disease

**Fig. 1:**
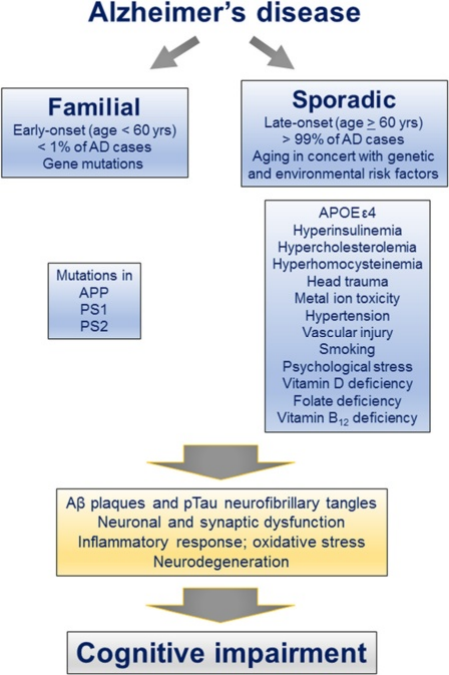
Multifactorial nature of AD and involvement of several different etiopathogenic mechanisms. Early-onset familial AD caused by mutations in APP, PS1, or PS2 constitutes < 1 % of AD cases. The exact causes of late-onset sporadic AD which accounts for the remaining > 99 % of AD cases are as yet largely unknown. However, aging alongside gene-environment interaction is speculated to contribute to this form of AD. Both forms of AD lead to amyloid plaque and neurofibrillary pathologies, synaptic dysfunction and neurodegeneration, and ultimately cognitive impairment

AD, first described by German psychiatrist Alois Alzheimer in 1907 [37], is a multifactorial and heterogeneous disease, and apparently involves several different etiopathogenic mechanisms (Fig. 1) [6, 38]. The early-onset familial form of AD which is caused by mutations in amyloid β precursor protein (APP), presenilin 1 (PS1), or presenilin 2 (PS2), accounts for <1 % of all cases (Fig. 1) (for review, [39, 40]). The exact causes of the late-onset sporadic form of AD, which accounts for over 99 % of the cases, are not yet understood. The sporadic form of AD probably involves several different etiopathogenic mechanisms in concert with aging such as metabolic derangements, head trauma, metal ion toxicity, hypertension, vascular injury, smoking, psychological stress, and vitamin deficiencies (Fig. 1) [38]. No known mutations are involved in sporadic form of AD, however, the presence of one or two apolipoprotein E epsilon 4 (APOE ε4) alleles increases the disease risk ~3.5-fold or ~10-fold, respectively [39–41].

**Fig. 2:**
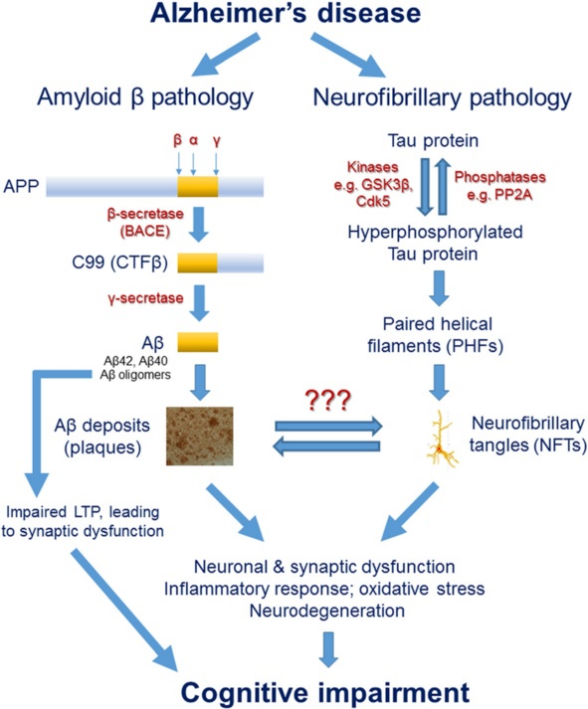
Aβ plaques and NFTs as hallmarks of AD. AD is characterized by extracellular deposits of Aβ (senile) plaques and intraneuronal NFTs leading to neurodegeneration, and ultimately cognitive impairment and dementia. Aβ plaques are produced by the amyloidogenic processing of APP by β- and γ-secretase enzymes leading to the formation of Aβ peptides (36–43 amino acids) of which the Aβ40 and Aβ42 are the most common. Aβ42 is the most fibrillogenic and thus most amyloidogenic Aβ peptide. Aβ oligomers impair hippocampal LTP, and thus synaptic plasticity, and learning and memory. NFTs are intracellular aggregates of MAP-tau which is abnormally hyperphosphorylated by upregulation of activities of kinases such as GSK3β and Cdk5 or deficit in phosphatases such as PP2A

The histopathological hallmarks in both the sporadic and the familial forms of AD are similar and include amyloid β plaques and NFTs leading to neurodegeneration (Fig. 2) [6]. The immensely popular amyloid cascade hypothesis suggests a major etiological role of Aβ for the NFT pathology, neurodegeneration and cognitive dysfunction in AD [42]. Aβ plaques are produced by amyloidogenic processing of the transmembrane protein, APP, by β- and γ-secretase enzymes resulting in the formation of major toxic products, Aβ40 and Aβ42 peptides (Fig. 2) [12, 43, 44]. The oligomeric form of Aβ has also been suggested as the main neurotoxic state of the peptide [45]. Aβ oligomers are known to inhibit hippocampal long-term potentiation (LTP) [46], a major cellular mechanism underlying synaptic plasticity, and learning and memory [47]. Amyloid cascade hypothesis also derives its major support from the Down syndrome individuals; as part of the trisomy 21, these patients carry 3 copies of APP and almost all of them develop AD neuropathological characteristics by the age of 40 years [48, 49].

Tau which is a neuronal microtubule associated protein (MAP) is a major protein subunit of paired helical filaments that constitute the NFTs (Fig. 2) [50, 51]. Tau in NFTs is abnormally hyperphosphorylated [13]. Tau is a substrate for several protein kinases including cyclin-dependent kinase 5 (Cdk5), glycogen synthase kinase 3 β (GSK-3β), and calcium/calmodulin activated protein kinase II (CaMKII) [52, 53]. The activities of several major tau kinases are regulated by protein phosphatase 2A (PP2A). PP2A can thus regulate the tau phosphorylaiton both directly and by inhibiting the activities of tau protein kinases [54]. PP2A activity is impaired and is possibly a cause of the abnormal hyperphosphorylation of tau in AD brain [55–57].

### Stages of Alzheimer’s disease

The neuropathologic staging of AD is performed based on three parameters (Aβ, Braak, and Consortium to Establish a Registry for AD, CERAD) to obtain an “ABC” score: histopathologic assessments of Aβ-containing amyloid plaques (A), Braak staging of NFTs (B), and scoring of neuritic amyloid plaques (C). The NFT load in AD brain are known to correlate better with cognitive decline [58]. In the earliest stages of AD (Braak stage I-II, prodromal AD), NFTs are confined to the entorhinal cortex [59, 60]. Subsequently, NFTs spread to the limbic and medial temporal lobe (Braak stage III-IV; mild cognitive impairment, MCI, or early AD); this stage correlates with early memory related symptoms in AD [61, 62]. During the late stages (Braak stage V–VI, clinical AD), the number of NFTs increase and they occur in neocortical areas leading to impairments in higher cognitive functions such as executive and visuospatial abilities, and speech in concert with AD-related cognitive deficits [60, 63].

**Fig. 3:**
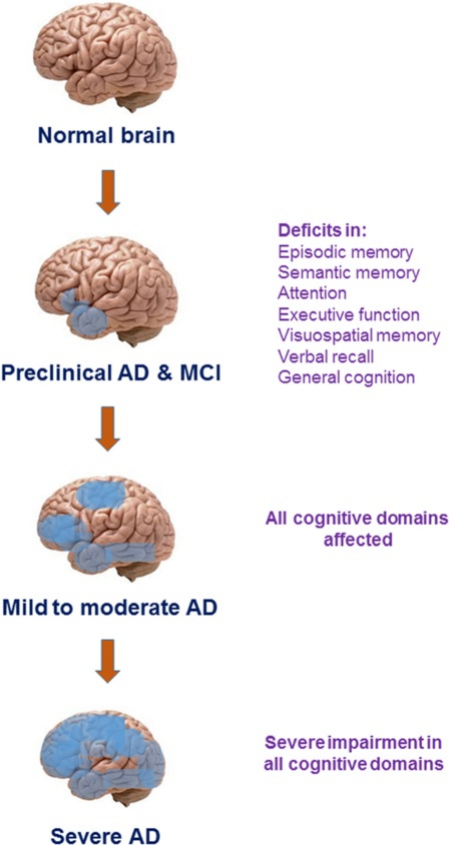
Progression of neuropathology and cognitive impairment in AD. The earliest AD neuropathology develops in medial temporal lobe and gives rise to episodic memory deficit followed by impairment in semantic memory (preclinical AD). As the patient progresses to MCI stage, higher cognitive domains such as attention, executive function, visuospatial memory, and verbal recall become impaired. Also, general cognition starts to decline. As the patient progresses to mild to moderate AD stages, the neuropathology spreads to frontal and parietal lobes, and all cognitive domains become affected. In severe stage of AD, the neuropathology spreads to involve all brain lobes, and a profound deficit in all cognitive domains is noted

AD is a progressive neurodegenerative disorder (Fig. 3). As mentioned before, the earliest neuropathological changes in AD are in the hippocampus and entorhinal cortex, followed by changes in the medial temporal lobe [64–68]. Correspondingly, the earliest detectable cognitive deficit is in the medial temporal lobe dependent episodic memory [62, 69, 70]. Episodic memory deficits are followed by impairment in semantic memory; both memory domains depend on the neural circuitry of medial and lateral temporal lobes and occur prior to deficit in higher cognitive functions such as attention, and executive and visuospatial capabilities [71]. Mild deficits in executive functioning are first detectable near the end of the preclinical phase of AD [72, 73]. As the patient progresses from preclinical stage of AD into MCI, more cognitive functions begin to be impaired including verbal recall and deterioration of general memory domain [71, 74]. As the patient progresses from MCI into mild, moderate, and subsequently the severe stages of AD, general cognition continues to decline with impairment manifesting in all cognitive domains [75].

### Neurogenic and synaptic failure in Alzheimer’s disease

Adult hippocampal neurogenesis plays a pivotal role in learning and memory [26–28, 30, 76]. The essential role of adult-born hippocampal neurons has been suggested in complex forms of spatial and associative memory [27, 77–81]. In rodents, the aberrant hippocampal neurogenesis leads to impairment in different forms of hippocampus dependent learning such as in Morris water maze and contextual fear conditioning tasks [82, 83]. AD and other neurodegenerative diseases are characterized by an imbalance between neurogenesis and neurodegeneration [18, 84]. Consequently, the impairment in neurogenesis has been hypothesized to contribute to learning and memory deficits in AD [17, 18, 84].

In adult hippocampus, the survival, maturation, and integration of new born neurons in the dentate gyrus depends on the appropriate *brain milieu* or microenvironment primarily provided by the neurotrophic factors [85–89]. The dentate gyrus microenvironment in neurodegenerative conditions such as AD is not conducive for the neurogenesis and the survival, maturation, and integration of new born neurons into the hippocampal functional circuitry [90–92]. The AD brain responds to neurodegeneration by stimulating neurogenesis, however, because of the lack of a proper neurotrophic microenvironment of the hippocampus, this effort of the AD brain to replace lost neurons with new neurons is unsuccessful and culminates in failure of neuronal survival, maturation, and integration [16, 17, 36, 93]. As the disease progresses, the neurogenic failure becomes severe, and contributes significantly to cognitive decline [18, 36].

AD has been described as a synaptic failure [22, 32]. AD brains also show dendritic and dendritic spine loss [94]. Quantitative studies on AD brains within 2–4 years after the clinically diagnosed disease showed a 25–35 % decrease in synaptic density and a 15–35 % synaptic loss per neuron in the frontal and temporal lobes of the cerebral cortex [15]. The extent of synaptic loss is even more severe in the hippocampus where it amounts to 44–55 % [19–21, 95, 96]. Remarkably, the synaptic loss in frontal cortex and limbic system is the best correlate of the severity of cognitive dysfunction [25, 33–35]. The profound neuronal, synaptic, and dendritic loss may contribute to impaired synaptic plasticity, including reduced LTP, in AD. Synaptic plasticity is known to be the cellular substrate of learning and memory [29]. Additionally, adult hippocampal neurogenesis has been proposed to play a pivotal role in synaptic plasticity, and subsequently, learning and memory processes in the hippocampus [97]. Both human AD cases and AD transgenic mice exhibit significant alterations in the process of adult hippocampal neurogenesis [16, 93, 98–104]. The synaptic plasticity impairments in AD, thus, may not only be the consequence of synaptic failure but also impaired neurogenesis.

### Shifting the balance from neurodegeneration to neural regeneration to treat Alzheimer’s disease

The final common outcome of various different etiopathogenic mechanisms involved in AD is neurodegeneration leading to cognitive impairment. Thus, a highly promising therapeutic strategy for AD is to shift the balance from neurodegeneration to neural regeneration [6, 36]. This can be achieved by utilizing means that can enhance adult hippocampal neurogenesis and neuronal and synaptic plasticity. Several different approaches have been employed in rodent models of AD with reasonable success to enhance neurogenesis and neuronal and synaptic plasticity [105]. Neural stem cells-based replacement approach for AD is currently the focus of intense research and has shown promising results in animal models of AD [106, 107]. Neural stem cell implantation in the triple transgenic AD mice (3xTg-AD) that harbor mutated human APP, tau, and PS1, has been reported to rescue cognitive impairment via increased brain derived neurotrophic factor (BDNF) expression [108]. Pharmacological stimulation of neural stem cells with neurotrophic factors and growth factors such as fibroblast growth factor 2 (FGF2) [109], BDNF [110], ciliary neurotrophic factor (CNTF) [111] has also shown promise in mouse models of AD.

### Neurotrophic factors --- potent neuronal and synaptic repair molecules for the treatment of Alzheimer’s disease

Neurotrophic factors can be broadly divided into 3 families: (1) neurotrophins, (2) glial cell-line derived neurotrophic factor (GDNF) family ligands (GFLs), and (3) neuropoeitic cytokines [112]. The neuroprotective effect of various neurotrophic factors via neuroregeneration and synaptic repair is well established [110, 113, 114].

The neurotrophin family comprises nerve growth factor (NGF), BDNF, neurotrophin 3 (NT3), and neurotrophin 4 (NT4) [115]. Neurotrophin signaling is mediated via two receptor types: p75 neurotrophin receptor (p75^NTR^) and tropomysin receptor kinase (Trk) [115]. Each Trk receptor selectively binds to different neurotrophins: NGF binds to TrkA, BDNF and NT4 bind to TrkB, and NT3 binds to TrkC [112, 115]. Neurotrophin signaling occurs via several different pathways including two major tyrosine kinase-mediated pathways: the phosphoinositide 3-kinase (PI3K)-AKT (also known as protein kinase B, PKB) pathway and the mitogen-activated protein kinase (MAPK)-extracellular signal-regulated kinase (ERK) pathway [112, 115]. The neurotrophin signaling mediates neuronal survival, proliferation, and differentiation [115]. Imbalance in the neurotrophin levels in different brain regions has been shown in AD cases [116]. BDNF levels have been shown to be decreased in AD brains suggesting a lack of trophic support that may contribute significantly to neurodegeneration [116–119]. BDNF is essential for basal level of adult hippocampal neurogenesis and also for the survival and integration of new-born neurons into the hippocampal circuitry [120, 121]. BDNF also plays a crucial role in both the early and late phases of LTP, the cellular substrate for learning and memory [122–125]. In mouse and primate models of AD, entorhinal cortex administration of BDNF was found to have a beneficial effect on cognition [110]. In human AD cases, decreased NGF levels have been shown in the nucleus basalis of Meynert [126], a group of neurons in the basal forebrain which has wide ascending projections to the neocortex, is rich in acetylcholine (Ach) and choline acetyltransferase (ChAT), and is well known to undergo degeneration in AD [127]. In animal models, it has been demonstrated that the ascending cholinergic projections in the brain express low- and high-affinity NGF receptors and are NGF-sensitive and probably NGF-dependent [128, 129]. Cholinergic degeneration leads to cognitive dysfunction, and treatment with NGF can improve cognition in animal models [128, 129]. NGF gene therapy has recently been shown to exert a neurotrophic effect in AD patients [130].

The GDNF family of ligands (GFL) comprises of four neurotrophic factors: GDNF, neurturin (NRTN), artemin (ARTN), and persephin (PSPN) [131]. GFL signaling occurs through binding of a particular GFL dimer to a cell surface bound co-receptor, a member of the GFRα protein family, and subsequent activation of a receptor tyrosine kinase molecule, RET (“rearranged during transfection”) [131]. The primary ligands for the co-receptors GFRα1, GFRα2, GFRα3, and GFRα4 are GDNF, NRTN, ARTN, and PSPN, respectively [131]. GFLs play a role in several different processes including neuronal survival, differentiation, and migration, and neurite outgrowth [131]. Deficiency of GFRα1, the receptor for GDNF, have been shown in human AD brains [132]. Also, GDNF gene therapy has been reported to protect against AD-like neuropathology in 3xTg-AD mice [133].

Neuropoietic cytokines family of neurotrophic factors exert many similar effects including enhancing neuronal proliferation and differentiation, however, this group signals through cytokine receptors rather than receptor tyrosine kinases [134, 135]. The transforming growth factor β (TGFβ) and interleukin-6 (IL-6) superfamilies are some of the most abundant and influential neuropoeitic cytokines in the brain, particularly during development [134, 135]. The TGFβ superfamily includes brain morphogenic proteins (BMPs) which play a key role in regulating neuron induction [134, 135]. BMPs interact with key neurogenic elements including Wnts and sonic hedgehog, and establish cell-fate determination and subtype differentiation [136–138]. The IL-6 family of cytokines includes CNTF, interleukin-11 (IL-11), leukemia inhibitory factor (LIF), oncostatin-M, cardiotrophin-1, and cardiotrophin-like cytokine [139, 140]. CNTF plays a pivotal role in adult hippocampal and subventricular zone neurogenesis, and the differentiation of neural stem cells [141–143]. CNTF signaling occurs through a tripartite complex of CNTF receptor α (CNTFRα), LIF β receptor (LIFR), and glycoprotein 130 (gp130). CNTF and LIF both signal through tyrosine phosphorylation (Tyr706) of the signal transducers and activators of transcription (STAT) proteins by the membrane associated Janus kinase (JAK) [144]. In the brain, CNTF is expressed in astrocytes in the neurogenic niches [145], while its receptor, CNTFRα, is expressed predominantly in neural progenitor cells and hippocampal neurons, and various other areas of the brain including motor cortex and cerebellum [142, 145]. Overall, CNTF is the most extensively studied neuropoetic cytokine, and its neuroprotective effects are well established [146]. CNTF administration has been shown to alleviate cognitive impairment and to stabilize synaptic protein levels in a transgenic mouse model of AD [111].

### Neurotrophic factor small-molecule mimetics for the treatment of Alzheimer’s disease

The therapeutic usage of neurotrophic factors such as BDNF and CNTF in AD patients is hindered by limited blood–brain barrier (BBB) permeability, poor plasma stability and unsuitable pharmacokinetics, and unwanted systemic effects [113, 147–149]. The recombinant BDNF or CNTF in clinical trials have shown limited bioavailability and multiple adverse effects [150, 151]. A promising strategy to bypass these limitations to develop neurotrophic factors based drugs for AD which has emerged during the last decade or so is to develop small-molecule mimetics that could exert the therapeutic beneficial effects of neurotrophic factors on neurogenesis, neuronal and synaptic plasticity, and ultimately cognition, with suitable pharmacokinetics and central nervous system (CNS) penetration for drug development, and without unwanted systemic effects produced by the full-length native molecules [148, 149, 152–156]. A major problem associated with the therapeutic usage of whole molecule neurotrophic factors is the pleiotropic actions that derive from their concomitant binding to multiple receptors [154]. The small-molecule mimetics might also allow to modulate various aspects of these signaling pathways in ways that are distinct from the whole molecule neurotrophic factors [154]. By departing from conventional neurotrophic factor signaling, these small-molecule mimetics might provide a novel therapeutic approach to treat AD [154].

Early attempts to develop small-molecule mimetics of neurotrophic factors focused on synthesizing peptidergic compounds consisting of amino acids residues corresponding to various NGF domains [154]. The first small peptide molecule corresponding to an NGF domain demonstrated to exert a neurotrophic effect was the cyclized dimeric form of peptide P7 (amino acid residues, KGKE) which acted through p75^NTR^ receptor [157]. The NGF small peptide mimetics containing KGKE or a homologous sequence have been reported to block Aβ binding to p75^NTR^ and protect against Aβ-induced cell death [158]. Another NGF small peptide mimetic, compound D3, which corresponds to amino acid side chains of NGF β-turn loops and acts through TrkA receptor, has been shown to rescue basal forebrain cholinergic neurodegeneration and spatial reference memory in aged rats [159]. Also, the administration of compound D3 via intracerebroventricular mini pump in J20 mice, which carry APP Swedish and Indiana mutations, has been shown to rescue learning and short-term memory deficits [160].

Small-molecule modulation of BDNF receptor, TrkB, for the treatment of neurodegenerative disorders including AD, has been the focus of intense research in recent years [113, 154, 161]. A naturally occurring flavone, 7,8-dihydroxyflavone (7,8-DHF), which can induce the phosphorylation of TrkB and its downstream mediators, AKT and ERK, has been shown to be neuroprotective and neurotrophic [161–163]. In studies employing transgenic mouse models of AD, 7,8-DHF has shown beneficial effect on learning and memory [164–166]. R7, a prodrug of 7,8-DHF, with considerably improved potency and favorable pharmacokinetics, is currently in preclinical development for the treatment of AD [167]. A BDNF small-molecule mimetic compound, LM22A-4, identified using *in silico* screening with a BDNF loop-domain pharmacophore, was found to promote survival of hippocampal neurons (~85 % of the efficacy of BDNF), induce TrkB phosphorylation, and prevent neuronal death in an in vitro model of AD [168]. Other TrkB small molecule mimetics that have shown promise in preclinical studies include de-oxygedunin [169], N-acetylserotonin [170], adenosine 2A receptor agonist [171, 172], and BDNF loop mimetics [173, 174]. Recently, our lab also identified BDNF tetrapeptides (peptide B1-B5) which can modulate TrkB signaling and exert neurotrophic effect in mouse hippocampal primary neuronal cultures [175].

A p75^NTR^ small-molecule non-peptide mimetic identified through *in silico* screening, LM11A-31, also known as C31, is a highly promising AD drug candidate [154]. LM11A-31 has been shown to block Aβ-induced deleterious signaling, including the activation of calpain-Cdk5, GSK-3β, and c-jun N-terminal kinase (JNK) signaling, and reduce Aβ-induced tau hyperphosphorylation and the inactivation of AKT and cAMP-response element-binding protein (CREB) [176]. LM11A-31 has also been reported to exert beneficial effect both at early and late stages of the AD-like neuropathology in transgenic mice [177, 178]. LM11A-31, has successfully completed a Phase I clinical trial without any significant adverse effects, and is currently undergoing Phase IIa clinical trial in human AD patients.

Neurotrophic factor small-molecule mimetics offer several advantages as compared to native neurotrophic factor molecules including suitable pharmacokinetics and enhanced BBB permeability for drug development. However, there are certain limitations such as insufficient receptor specificity, requirement for continuous dosing, and effects which are not brain region-specific [154]. These limitations need to be taken into consideration for neurotrophic factor small-molecule mimetics based drugs [154].

### CNTF small-molecule peptide mimetic, P021, as a drug candidate for Alzheimer’s disease

As mentioned before, the neuroprotective effects of CNTF are well established [146], and it has been reported to rescue cognitive dysfunction in AD transgenic mice [111]. However, like other neurotrophic factors, the clinical therapeutic usage of CNTF is restricted due to its short plasma half-life and severe adverse effects including anorexia, skeletal muscle loss, muscle pain and cramps, and hyperalgesia, experienced upon peripheral administration in humans [150].

**Fig. 4:**
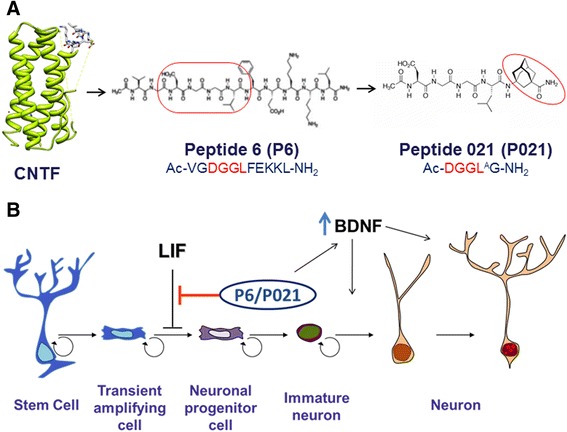
Design and structures of CNTF small-molecule mimetics and their mechanism of action to enhance formation of NPCs and mature neurons. **a** Protein Data Base rendering of one 4-helix bundle of truncated human CNTF (Residues 2–187), generated from CNTF. Only one protein chain is shown for clarity. Residues 149GGLFEKKL156 are shown as a tube model, while the rest of the sequence are presented as ribbon [98]. The structures of P6 and P021 are also shown. From the neurogenic 11-mer, Ac-VGDGGLFEKKL-NH_2_ (P6), a truncated, still neurogenic pentamer, with an adamantylated glycine group (red oval), Ac-DGGLA^G^-NH_2_ (P021) was designed [191]. **b** P6 and P021 enhance neural progenitor cells (NPCs) formation and maturation and integration of new-born neurons by competitively inhibiting the LIF signaling and increasing BDNF expression respectively [99, 180, 181, 191, 192]

Our laboratory generated an 11-mer CNTF small-molecule peptide mimetic, Peptide 6 (P6; Ac-VGDGGLFEKKL-NH_2_), which corresponds to a biologically active region (amino acid residues 146–156) of human CNTF and was identified using epitope mapping and employing neutralizing antibodies (Fig. 4) [179, 180]. P6 has a plasma half-life of over 6 h as compared to ~ 3 min plasma half-life of recombinant CNTF, and is BBB permeable [180]. P6 acts by competitively inhibiting the LIF signaling, thus promoting the formation of neural precursor cells (NPCs), and by increasing BDNF mRNA levels, thus increasing the survival, maturation, and integration of new-born cells (Fig. 4) [180, 181]. Peripheral administration of P6 for 30 days via a subcutaneously implanted slow release bolus, was found to enhance dentate gyrus neurogenesis, neuronal plasticity, and spatial memory of the normal adult C57Bl/6 mice [180]. The intraperitoneal administration of P6 for six weeks in 6–7 month-old 3xTg-AD mice (at early stages of the AD pathology prior to Aβ plaques and neurofibrillary tangle-like pathology) ameliorated impairment in spatial reference memory and short-term episodic memory by enhancing dentate gyrus neurogenesis and neuronal plasticity in these mice [98]. The 3xTg-AD mice carry AD-related mutation including human PS1 M146V, human APP Swedish, and human tau P301L [182], and develop Aβ plaques and neurofibrillary tangle-like pathologies in a progressive and age-dependent manner, starting at ~9 and ~12 months respectively but show cognitive impairment as early as 3–5 months of age [182–185]. P6 treatment in 6–7 month-old 3xTg-AD mice could not exert any effect on Aβ and tau pathologies [98], seen in this age mice as intraneuronal Aβ accumulation and tau hyperphosphorylation, and not as plaques and tangles [87, 183]. Other preclinical studies employing P6 showed neurogenic and neurotrophic effects in animal models of sporadic AD [181], familial AD [186], Down syndrome [187], autism [188], and traumatic brain injury [189].

To increase the “drug-like” pharmacokinetic properties, we narrowed down the minimal active region of P6 to 4 amino acid residues (Ac-DGGL-NH_2_, Peptide 6c, P6c) which could still exert neurogenic and neurotrophic effects [190]. We subsequently added adamantylated glycine group to P6c to enhance its BBB permeability, and the resultant pentamer (Ac-DGGL^A^G-NH_2_), called Peptide 021 (P021) (Fig. 4), was found to enhance proliferation and differentiation of adult hippocampal progenitors, increase synaptic markers expression, and improve cognition in C57Bl/6 mice [191]. Oral administration of the compound P021 could rescue cognitive aging by enhancing neurogenesis via increased BDNF expression and by decreasing tau levels in aged Fisher rats [192, 193].

**Fig. 5:**
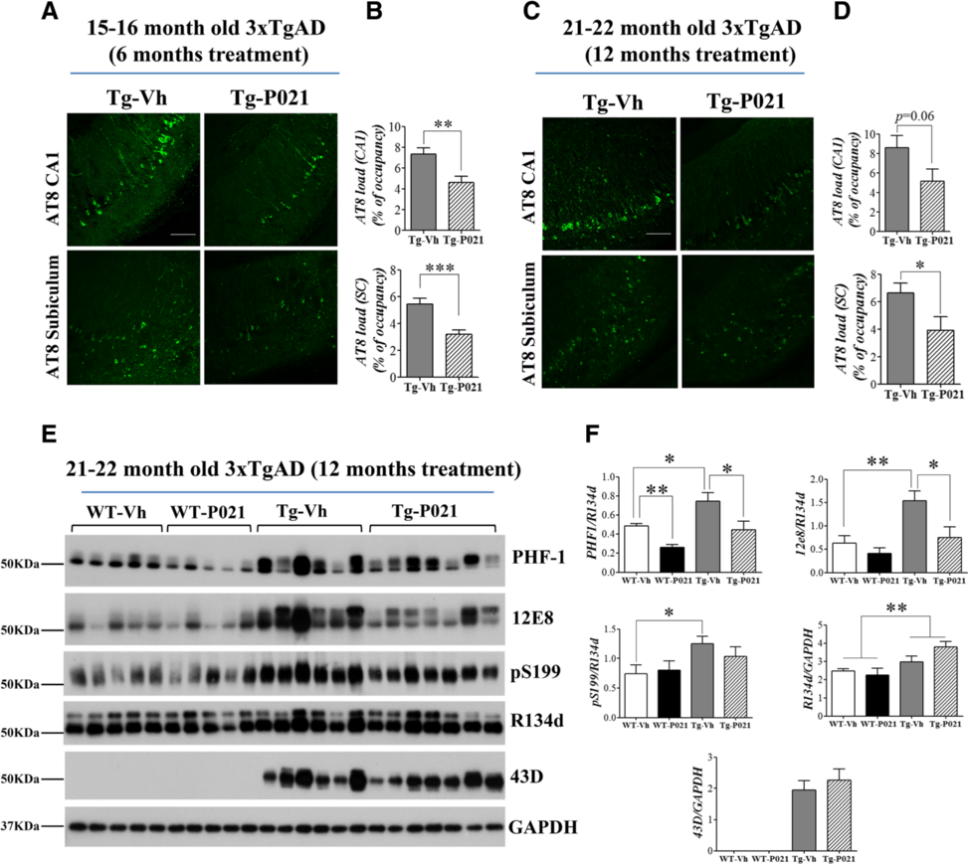
P021 treatment reduces abnormal hyperphosphorylation of tau in 3xTg-AD mice. Reprinted from Kazim et al. [99] with permission from Elsevier. **a**-**d** In the subiculum and the CA1 regions of the hippocampus, AT8 (phospho-tau: pSer202, pThr 205) load was significantly reduced by P021 treatment in 3xTg-AD mice (both 15–16 month-old/6 months treatment group and 21–22 month-old/12 months treatment group). **a** Representative photomicrographs illustrating AT8 immunoreactivity in the different regions of hippocampus from the 15–16 month-old/6 months treatment group are shown. **b** The AT8 load was calculated as the percentage of area occupied by immunoreactive label. Quantification of the immunoreactivity is shown as mean ± S.E.M. from Tg-Vh (*n* = 7), and Tg-P021 (*n* = 7). **c** Representative photomicrographs illustrating AT8 immunoreactivity in the different regions of hippocampus from the 21–22 month-old/12 months treatment group are shown. **d** The AT8 load calculated as the percentage of area occupied by immunoreactive label is shown as mean ± S.E.M. from Tg-Vh (*n* = 6), and Tg-P021 (*n* = 6). **e**, **f** Western blot analyses of tau pathology in 21–22 month old (12 months treatment) group. P021 treatment significantly reduced abnormal hyperphosphorylation of tau at sites pSer396/pSer404 (PHF-1) and pSer262/pSer356 (12E8). Pan-tau antibody, R134d did not show any significant difference between groups. Blots developed with human specific tau antibody 43D showed the protein expression only in 3xTg-AD mice. Quantification of the Western blots is shown as mean ± S.E.M. from WT-Vh (*n* = 5), WT-P021 (*n* = 5), Tg-Vh (*n* = 6), and Tg-P021 (*n* = 7). *p < 0.05, **p < 0.01, and ***p < 0.001. *Scale bar* = 100 μm. WT-Vh = wild-type control mice treated with vehicle diet; WT-P021 = wild-type mice treated with P021 diet; Tg-Vh = 3xTg-AD mice treated with vehicle diet; Tg-P021 = 3xTg-AD mice treated with P021 diet

**Fig. 6:**
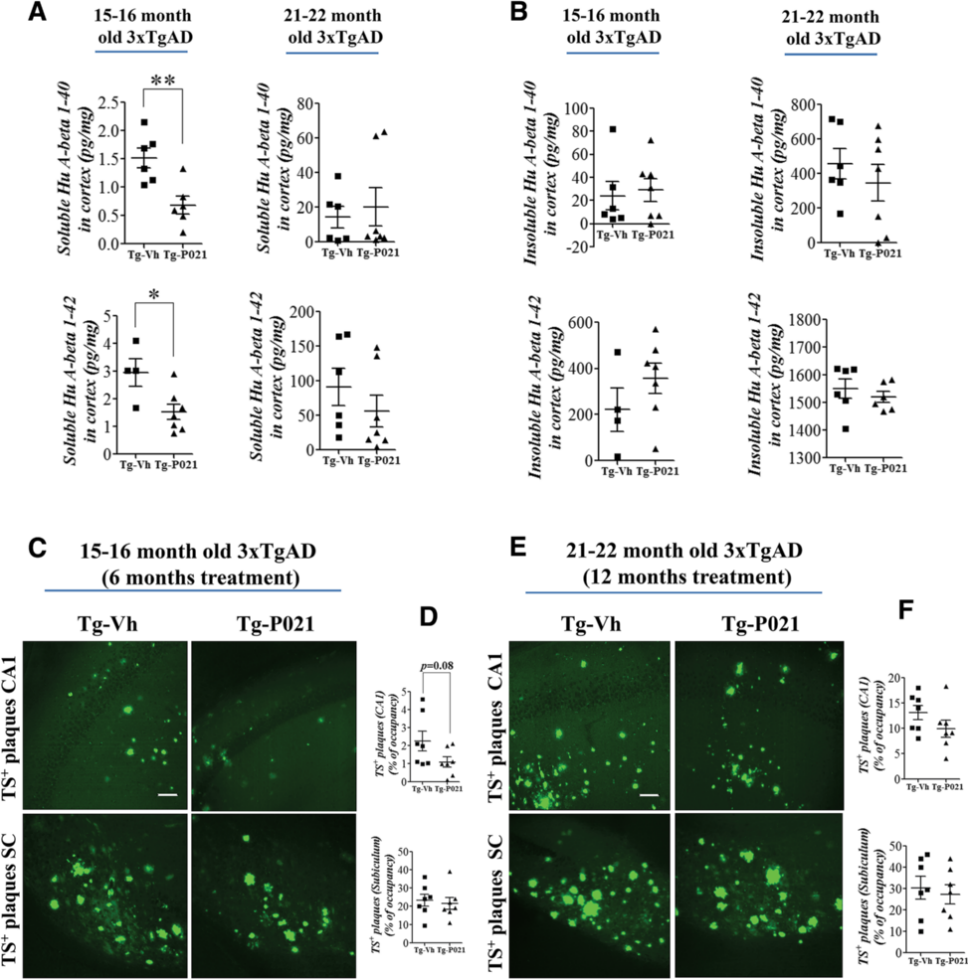
P021 treatment reduces soluble Aβ in 3xTg-AD mice. Reprinted from Kazim et al. [99] with permission from Elsevier. **a**, **b** ELISA quantification of soluble and insoluble Aβ 1–40 and Aβ 1–42 in the cortex revealed significant reduction of soluble Aβ with P021 treatment in 15–16 month-old/6 months treatment group but not in 21–22 month old/12 months treatment group. No effect on insoluble Aβ was found. Quantification is shown as mean ± S.E.M. from Tg-Vh (*n* = 5–7) and Tg-P021 (*n* = 6–7). **c**, **d** Representative photomicrographs illustrating thioflavin S (TS)^+^ plaque load in the CA1 and subiculum regions of the hippocampus from 15–16 month old/6 months treatment mice are shown. Quantification of TS^+^ load is shown as mean ± S.E.M. from Tg-Vh (*n* = 7), and Tg-P021 (*n* = 7). **e**, **f** Representative photomicrographs illustrating TS^+^ plaque load in the CA1 and subiculum regions of the hippocampus from 21–22 month old/12 months treatment mice are shown. Quantification of TS^+^ load is shown as mean ± S.E.M. from Tg-Vh (*n* = 7), and Tg-P021 (*n* = 6–7). *p < 0.05, **p < 0.01. *Scale bar* = 100 μm

**Fig. 7:**
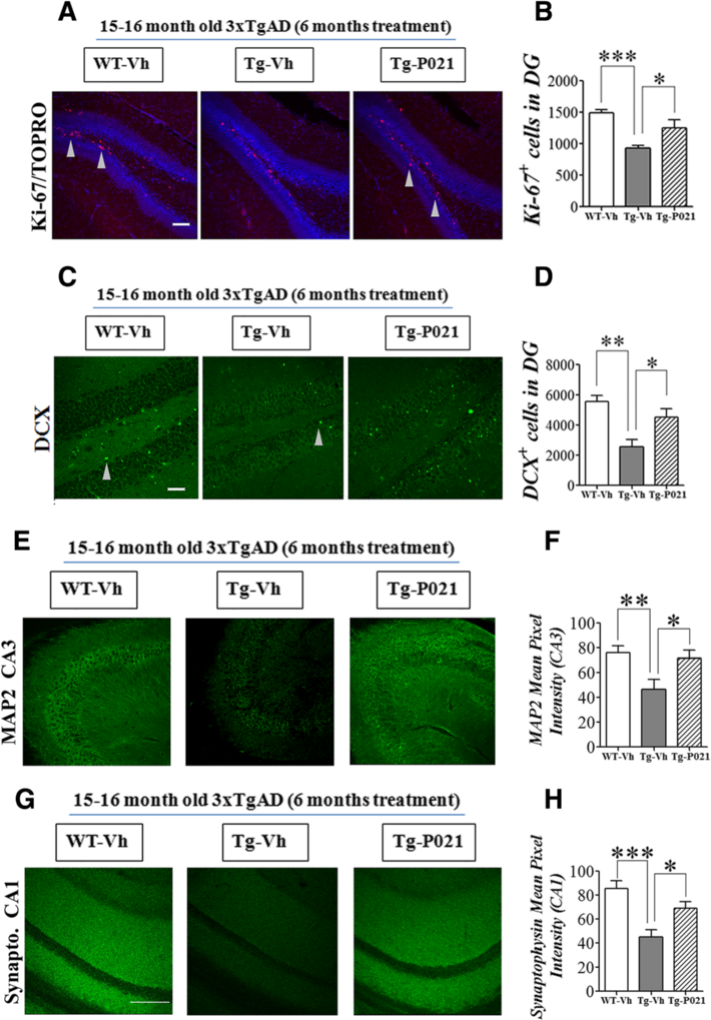
P021 treatment enhances dentate gyrus neurogenesis and rescues dendritic and synaptic loss in 3xTg-AD mice. Reprinted from Kazim et al. [99] with permission from Elsevier. **a**-**d** DG neurogenesis, as evaluated by counting Ki-67 (cell proliferation maker) and doublecortin (DCX, marker for immature neurons) positive cells, was significantly deficient in 15–16 month-old 3xTg-AD mice, and was corrected by P021 treatment. **a**, **c** Representative photomicrographs illustrating Ki-67+/TOPRO and doublecortin (DCX) + cells in the DG of hippocampus from 15–16 month-old/6 months treatment mice. **b**, **d** Densitometric quantification data of Ki-67+ and DCX+ cells are shown as mean ± S.E.M. from WT-Vh (*n* = 6), Tg-Vh (*n* = 7) and Tg-P021 (*n* = 7), and WT-Vh (*n* = 5), Tg-Vh (*n* = 6), and Tg-P021 (*n* = 6), respectively. Arrow heads indicate positive cells. **e**-**h** The 15–16 month old 3xTg-AD mice showed significantly reduced MAP2 (dendritic marker) and synaptophysin (presynaptic marker) expression level (fluorescence intensity) in the CA3 and CA1 regions of the hippocampus, respectively. P021 treatment significantly ameliorated this deficit. **e** Representative photomicrographs illustrating MAP2 immunoreactivity in the CA3 region. **f** Densitometric quantification of the immunohistochemistry is shown as mean ± S.E.M. fromWT-Vh (*n* = 6), Tg-Vh (*n* = 7), and Tg-P021 (*n* = 7). **g** Representative photomicrographs illustrating synaptophysin immunoreactivity in the CA1 region. **h** Densitometric quantification of the immunohistochemistry is shown as mean ± S.E.M. from WT-Vh (*n* = 6), Tg-Vh (*n* = 6), and Tg-P021 (*n* = 6). *p < 0.05, **p < 0.01, and ***p < 0.001. *Scale bar* = 100 μm

**Fig. 8:**
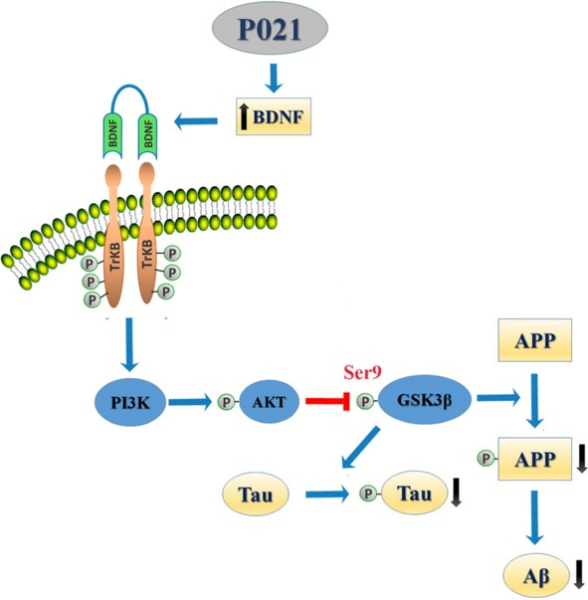
Mechanism of the disease-modifying effect of P021 in AD. The CNTF small-molecule peptide mimetic compound, P021, increases BDNF expression which via the TrkB-PI3K-AKT pathway leads to inhibitory phosphorylation of GSK3β at Ser9 [99]. GSK3β is major tau kinase and contributes to abnormal hyperphosphorylation of tau at several major sites, and also plays a role in the amyloidogenic processing of APP. P021, via decreasing the GSK3β activity, causes decrease in tau and Aβ pathologies

In a recent study, we treated 3xTg-AD mice with P021 in diet (60 nmol/g feed) starting at 9–10 months of age (early-moderate stages of the AD-like neuropathology) for 12 months, and evaluated the therapeutic effect of the compound at 15–16 months of age (moderate-severe pathology) and at 21–22 months of age (severe pathology) [99]. P021 treatment significantly reduced tau pathology both at moderate and severe stages of the pathology in 3xTg-AD mice (Fig. 5), however, the effect of P021 on Aβ pathology was limited to a significant decrease in soluble Aβ levels and a trend towards reduction in Aβ plaque load in CA1 region of hippocampus, consistent with reduction in Aβ generation and not clearance (Fig. 6) [99]. P021 treatment also rescued cognitive impairment, enhanced dentate gyrus neurogenesis, and ameliorated synaptic deficit at moderate to severe stage of the pathology in 3xTg-AD mice (Fig. 7). The effect of P021 on tau and probably also on Aβ pathology was via increased BDNF expression mediated activation of TrkB-PI3K-AKT signaling pathway which leads to down-stream inhibition of GSK-3β activity by increase in its inhibitory phosphorylation at Ser9 by AKT (Fig. 8) [99, 194]. GSK3β is a major tau serine/threonine kinase which is known to phosphorylate tau at many different sites including Ser199, Ser202, Thr205, Ser396, and Ser404 [52, 53]. Reduction in GSK3β activity has also been demonstrated to ameliorate Aβ pathology via reduction in amyloidogenic processing of APP [195, 196].

The compound P021 has favorable pharmacokinetics for drug development including plasma half-life of >3 h and stability of >95 % and >90 % in artificial intestinal fluid up till 2 h and in artificial gastric juice up till 30 min, respectively, and is BBB permeable [99]. Up to 1 year of P021 administration did not induce any adverse effect in 3xTg-AD mice or in wild type control [99].

### Conclusions

AD is a multifactorial and heterogeneous devastating neurodegenerative disorder which contributes significantly to health care burden worldwide, more so because of the lack of an effective therapy. Independent of the various etiopathogenic mechanisms involved in AD, the neurogenic and synaptic failure are a common feature of AD that play a pivotal role in cognitive dysfunction. The rationale for the use of neurotrophic factors based approach is quite strong by virtue of their well-established pro-neurogenic effects and synaptic repair potential. However, the unfavorable pharmacokinetics, poor brain penetration, and undesired adverse effects associated with the native neurotrophic factor molecules limit their clinical usage. Neurotrophic factor small-molecule mimetics offer a highly promising strategy to overcome these limitations for AD drug development. Preclinical studies have demonstrated therapeutic beneficial effects of several neurotrophic factor small-molecule mimetics particularly the BDNF and CNTF mimetics. These compounds are currently undergoing or will undergo in near-future human clinical trials to assess their therapeutic efficacy in human AD patients. Overall, the neurotrophic factor small-molecule mimetics based neuroregeneration and synaptic repair represents a highly promising therapeutic strategy for AD, and can lead to the development of an effective drug to rescue cognitive impairment.

## Abbreviations

3xTg-AD, triple transgenic Alzheimer’s disease mice; 7,8-DHF, 7,8-dihydroxyflavone; Ach, acetylcholine; AD, Alzheimer’s disease; APOE ε4, apolipoprotein E ε4; APP, amyloid β precursor protein; ARTN, artemin; Aβ, amyloid β; BBB, blood–brain barrier; BDNF, brain-derived neurotrophic factor; BMP, bone morphogenic protein; CaMKII, calcium/calmodulin activated protein kinase II; Cdk5, cyclin-dependent kinase 5; CERAD, consortium to establish a Registry for Alzheimer’s disease; ChAT, choline acetyltransferase; CNS, central nervous system; CNTF, ciliary neurotrophic factor; CNTFRα, ciliary neurotrophic factor receptor α; CTFβ, C-terminal fragment β; ERK, extracellular signal-regulated kinase; FGF2, fibroblast growth factor 2; GDNF, glial cell-derived neurotrophic factor; GFL, glial cell-derived neurotrophic factor family ligands; GFRα, glial cell-derived neurotrophic factor family receptor alpha; gp130, glycoprotein 130; GSK-3β, glycogen synthase kinase 3 β; IL-11, interleukin-11; IL-6, interleukin-6; LIF, leukemia inhibitory factor; LIFR, leukemia inhibitory factor β receptor; LTP, long-term potentiation; MAP, microtubule associated protein; MAPK, mitogen-activated protein kinase; MCI, mild cognitive impairment; NFTs, neurofibrillary tangles; NGF, nerve growth factor; NPCs, neural precursor cells; NRTN, neurturin; NT3, neurotrophin 3; NT4, neurotrophin 4; P021, peptide 021; P6, peptide 6; p75^NTR^, p75 neurotrophin receptor; PI3K, phosphoinositide 3-kinase; PKB/AKT, protein kinase B; PP2A, protein phosphatase 2A; PS1, presenilin 1; PS2, presenilin 2; pSer, phosphorylated serine; PSPN, persephin; pTau, abnormally hyperphosphorylated tau protein; pThr, phosphorylated threonine; RET, rearranged during transfection; TGFβ, tansforming growth factor β; Trk, tropomysin receptor kinase.
